# Children’s participation in the development, use and evaluation of support interventions for children of a parent diagnosed with cancer: a scoping review protocol

**DOI:** 10.1136/bmjopen-2024-084240

**Published:** 2024-08-28

**Authors:** Maria Samuelsson, Marie-Louise Möllerberg, Kristina Edman, Kristofer Hansson, Karin Enskär, Anne Wennick

**Affiliations:** 1 Department of Care Science, Faculty of Health and Society, Malmo University, Malmo, Sweden; 2 Skanes universitetssjukhus Malmo, Malmo, Sweden; 3 Department of Social Work, Malmo University Faculty of Health and Society, Malmo, Sweden; 4 Department of Women's and Children's Health, Uppsala Universitet, Uppsala, Sweden

**Keywords:** Family, ONCOLOGY, Psychosocial Intervention, Child, Review

## Abstract

**Abstract:**

**Introduction:**

At times of parental cancer, children’s health and well-being are at risk, which is why interventions to support these children have been developed. When developing such interventions, engagement of the population under study in research is endorsed to enhance relevance of research questions and to enhance uptake and dissemination of the findings. Since no previous review has mapped the ways children participate in the development, use and evaluation of these support interventions, the focus of the upcoming scoping review is to identify gaps in the literature for guidance of future research.

**Methods and analysis:**

The scoping review is guided by the methodological framework developed by Arksey and O’Malley. A preliminary search strategy was performed in PubMed in November 2020, refined in March 2021 and applied in PubMed, PsycINFO and CINAHL. Additional searches were performed in Google Scholar and SwePub, and reference lists were hand searched. Refined searches will be conducted in February 2024. The multidisciplinary research team will independently screen titles, abstracts and full-text articles for relevance. Then, relevant studies will be critically evaluated using the Joanna Briggs Critical Appraisal Skills Tools. Data will be extracted using an extraction form and analysed deductively. A descriptive summary of study characteristics and the research process will be presented, including a flow chart. The reporting of the study will be guided by the Preferred Reporting Items for Systematic Reviews and Meta-Analyses Extension for Scoping Reviews Checklist.

**Ethics and dissemination:**

Being a secondary analysis, ethical approval is not needed. Still, relevant studies will be reviewed for ethical approval as a criterion for inclusion. The findings will be used to inform future studies and will be published in a scientific journal as well as presented at conferences and organisations for children’s rights.

**STRENGTHS AND LIMITATIONS OF THIS STUDY**

- Search strategies were developed in collaboration with a research librarian.
- Reporting adheres to the relevant EQUATOR guidelines.
- Multidisciplinary researchers will screen each record’s potential for inclusion.
- Only literature in English and Swedish will be included.

## Introduction

 It is well known that severe parental illness, such as parental cancer, is a risk factor for several negative consequences in a child’s life.^1 2^ Parental cancer is, for instance, adversely associated with school absenteeism, increased healthcare needs and negative effects on mental health.^3^ In parallel, multiple supportive care needs have been identified among children of parents diagnosed with cancer,^4^ for example, the children’s need for age-appropriate information about their parent’s cancer, the need for help in communicating with their parents and family and the need for peer support to normalise their feelings and reduce feelings of isolation. Corresponding with these needs and the evidence on the short-term and long-term effects on children’s lives, actions from a care science and social work perspective have been taken to support these children.^4^,^7^

Several reviews have been undertaken that focus on support for children of a parent diagnosed with cancer,^4^,^7^ yet from different perspectives. Ellis *et al*^4^ evaluated psychosocial interventions^4^ whereas Ohan *et al*^6^ focused on which needs the support intervention addresses. Niemelä *et al*^7^ reviewed child-centred interventions, with a focus on prevention. Moreover, Inhestern *et al*^5^ reviewed psychosocial support for children of parents diagnosed with cancer from the perspective of intervention development, evaluation and implementation, with special attention on hinders for implementation of support interventions for children in clinical care. Despite these comprehensive reviews, there are still unanswered questions regarding children’s participation in these interventions. Due to a paradigm shift in child health research, from a family-centred viewpoint to a child-centred viewpoint,^8^ this study focuses on the existing literature from a child-centred viewpoint. Namely, it focuses on children’s participation in the development, use and evaluations of the interventions, that is, both in research processes and as study participants. In child health research, children can participate indirectly or directly, for example, through being present or being engaged. For this study, participation is defined in accordance with Eriksson and Granlund^9^ as “a feeling of belonging and engagement experienced by the individual in relation to being active in a certain context” and divided into two dimensions: presence (ie, physically being there) and engagement (ie, expressions of involvement). Thus, this scoping review will aim to provide an overview of the ways children participates (directly or indirectly) in the development, use and evaluation of the support interventions by mapping literature on support interventions for children of parental cancer including children as study participants.

The rationale for this study is the growing awareness of the importance of patient and public involvement in research (PPI-R).^10^,^13^ PPI-R is defined as research to being carried out ‘with’ or ‘by’ members of the public, rather than ‘to’, ‘about’ or ‘for’ them. In line with this, the UN Convention on the Rights of the Child^14^ states that, in all situations that concern children, children have the right to have their voices heard. Arguably, this statement also applies to research. Involving the population under study is endorsed to ensure that those with a lived experience of the phenomenon being researched—those who are the most affected by the research findings—are listened to and can contribute to decision-making in the research processes, for example, through the formulating and prioritising of research questions and the strategies and timings for data collection.^15^ In addition, reported benefits are improved recruitment and response and retention rates,^16^ more relevant research and the enhanced uptake and dissemination of the findings.^17^ Thus, the upcoming scoping review aims to map the existing literature in the field of support interventions for children of parental cancer in search for how children are involved. By conducting a scoping review, gaps in the literature can be identified to direct further research in purpose of developing support interventions that correspond with the children’s needs and preferences. In preparation for the scoping review, searches were made to locate a comparable, published or ongoing, systematic and/or scoping review in PubMed, PsychINFO, Cumulative Index to Nursing and Allied Health Literature (CINAHL), Cochrane Library and PROSPERO. However, none were identified.

### Aim

The purpose of the scoping review is to map the existing literature on children’s participation in the development, use and evaluation of support interventions for children of a parent diagnosed with cancer.

## Method

This protocol is developed from the Preferred Reporting Items for Systematic Reviews and Meta-Analysis Protocols (PRISMA-P).^18^ The protocol refers to an upcoming scoping review that will be undertaken iteratively following the six stages first established by Arksey and O’Malley^19^ and later refined by Levac *et al*^20^ and Colquhoun *et al*,^21^ all of which are described by the Joanna Briggs Institute (JBI)^22^: (1) identifying the research question, (2) identifying relevant studies, (3) selecting studies, (4) charting the data, (5) collating, summarising and reporting the results, and (6) consultation. Review registration osf.io/yf36w.

A scoping review was chosen because it allows for a broad mapping of a research field to direct further inquiry. Therefore, a variety of study designs will be included as disparate or heterogeneous sources, are according to Arksey and O’Malley,^19^ useful for bringing together evidence from and enabling the identification of gaps in the literature. Additionally, the reporting of the study will be guided by the PRISMA Extension for Scoping Reviews Checklist by Tricco *et al*.^23^ To achieve rigour, enable replication and enhance reliability, respectively, the search and selection process will be illustrated in a flow chart, PRISMA for scoping reviews.

### Stage 1: identifying the research question

Given that the aim of this study was broad in nature, a Participants, Concept, and Context mnemonic was developed (table 1) in accordance with the JBI.^22^ Based on its criteria and determinants, the following overarching research question was formulated: What is known from the existing literature on children’s participation in the development, use and evaluation of support interventions for children of a parent diagnosed with cancer? For this study, a child is a person <18 years,^14^ and parents are defined as biological parents or legal guardians.

**Table 1:** The Population Concept and Context mnemonic, as recommended by Joanna Briggs Institute

Criteria	Determinants
Participants	Children
Concept	Support
Context	Parental cancer

The specific research questions are as follows:

1. If, how and when do the children participate in the intervention development (directly or indirectly, eg, by proxy)?
2. How does the children use the intervention (directly or indirectly, eg, by proxy)?
3. If, how and when does children’s participate in evaluations of the intervention (directly or indirectly, eg, by proxy)?

These derive from the definition of participation^9^ applied.

### Stage 2: identifying relevant studies

In November 2020, a search strategy was designed by the multidisciplinary research team of Registered Nurses (MS, M-LM, KE, and AW) and Registered Sick Childrens’ Nurses (MS, KE and AW), Registered Healthcare Counsellor (KE) and Ethnologist (KH), and further developed in collaboration with an experienced librarian well versed in research databases. Subsequently, a draft search with a preliminary search strategy was conducted in PubMed and PsychINFO (online supplemental file 1). The overall result was that no limitations were set regarding study design. Words in the title and abstracts, as well as index terms describing the papers were then analysed by two members of the research team (MS and AW), resulting in a revised search strategy that was applied in March 2021 in the following databases: PubMed, CINAHL, Cochrane Library and PychINFO to cover a comprehensive sample of literature concerning healthcare. In accordance with Arksey and O’Malley’s^19^ recommendation of an iterative search process, a second analysis will be undertaken before initiating refined searches in all databases reported above as well as in Web of Science. Search tools such as Medical Subject Headings, Headings, Thesaurus and Boolean operators (AND/OR) will be used to expand and narrow the search and keywords, for example, ‘cancer’ and ‘neoplasm’ and synonyms of, for example, ‘experience’, ‘perception’ and ‘nuanced’ will be applied to the different databases. Lastly, the reference lists of retrieved articles will be hand searched for additional studies. Grey literature will also be searched for references, but not included unless they are research studies. The inclusion criteria are primary studies, regardless of design, published in Swedish or English in peer-review journals focusing on

1. support to children aged 0–18 years,
2. a parent diagnosed with or deceased or cured from cancer.

Studies will be excluded if children aged 0–18 years are not identifiable or if the extraction of qualitative or quantitative data with children aged 0–18 years is not possible. Additionally, to enable the systematic exclusion of studies with incomplete methodological description, the JBI Critical Appraisal Tools will be used to evaluate the quality of included studies. In general, the quality of a study is not assessed in scoping reviews.^19^ However, the eligible full-text articles will be assessed for incomplete methodological description. Therefore, a cut-off point will be set at studies not presenting the following: aim, criterions for inclusion and exclusion, participants, data collection and the process of analysis. These studies will be categorised as ‘low quality’. Studies not selected for inclusion will be documented with the reason(s) for exclusion in a separate file.

### Stage 3: study selection

**Figure 1:**
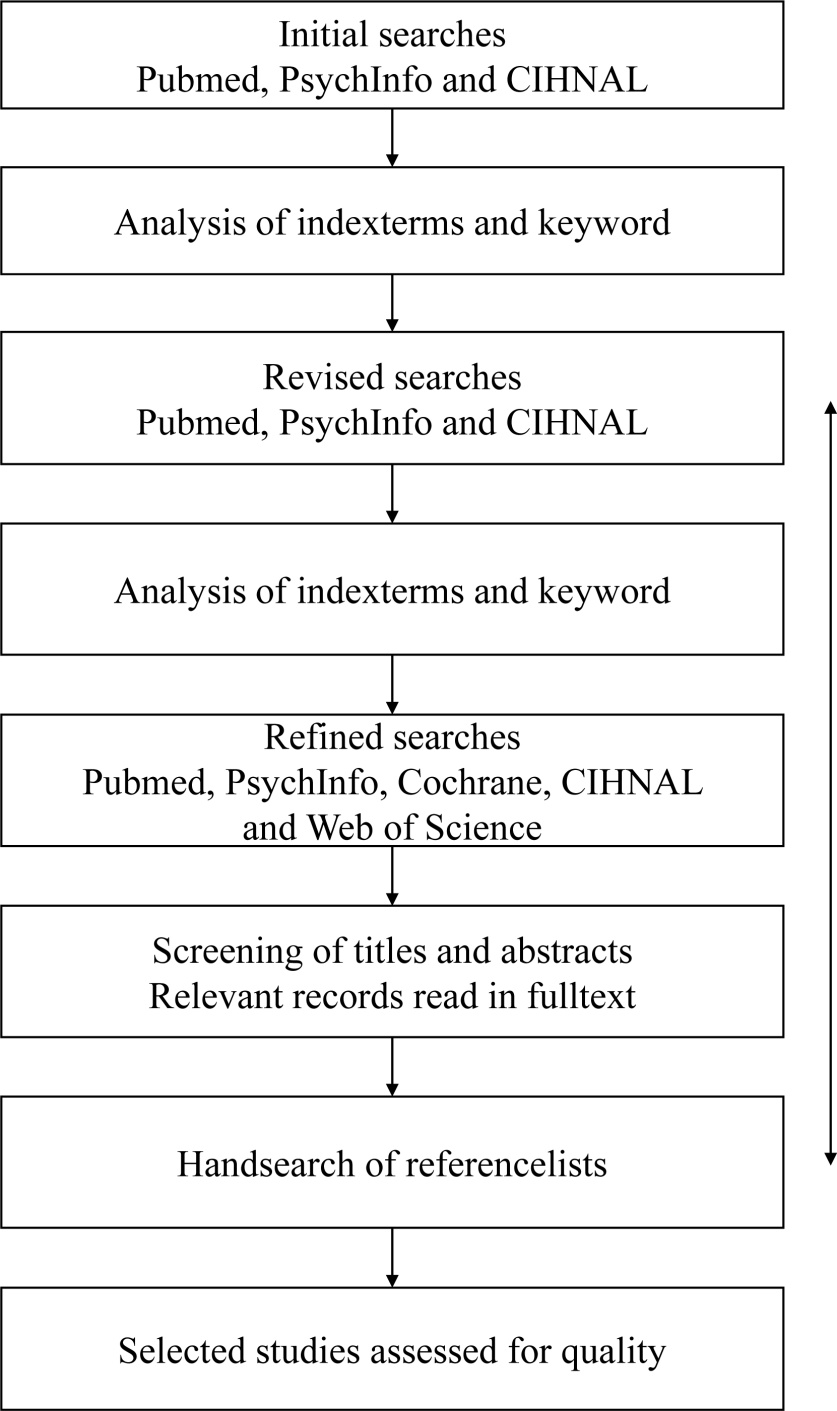
Flow chart of the search and selection process. CINAHL, Cumulative Index to Nursing and Allied Health Literature.

Members of the research team will screen the identified studies for relevance. To enhance cohesion, two members will screen all titles and abstracts, respectively, followed by a discussion among the researchers. Yet another member will be assigned to resolve possible disagreements. Next, the abstracts will be divided among all members in the research team and assessed in pairs (one-third per pair). Again, one member will be assigned to resolve possible disagreements. Both the screening of titles and abstracts will be conducted using the Rayyan system for systematic reviews.^24^ The system enables blinded screening by multiple researchers. Eligible studies will be retrieved and read in full, followed by quality assessments. This step will be undertaken by two researchers who independently assess each record for relevance and quality, followed by discussion, as mentioned. Figure 1 shows an overview of the planned search and selection process.

### Stage 4: charting the data

In accordance with JBI,^22^ a data extraction form will be developed to extract the study characteristics and findings that match the aims of the review. The following elements are planned to be extracted:

1. Context.
2. Study characteristics.
3. Participant characteristics.
4. Intervention characteristics.
5. Intervention development.
6. Use of the intervention.
7. Intervention evaluation.
8. Intended outcomes.

The research team will pilot the extraction on three articles and cross-check for reliability.

In line with the methodology, the charting process is also an iterative process**,** meaning the data extraction form may be modified. If so, this will be described in the final manuscript.

### Stage 5: collating, summarising and reporting the results

A descriptive summary of the scoping review process and of study characteristics will be presented. This stage will be segmented into the three steps, as suggested by Levac *et al*^20^: analysing the data; reporting the results linked to the research question; and interpreting the implications of the results for research, policy and practice. In accordance with the methodology described by JBI,^22^ extracted data will be analysed using descriptive content analysis as outlined by Elo and Kyngäs,^25^ guided by specific research questions divided under the headings ‘Development’, ‘Use’ and ‘Evaluation’. The result will be presented through a narrative summary of the included articles and findings complemented with tables and figures when appropriate.

### Stage 6: consultation

To enhance the methodological rigour of the final result, preliminary findings will be presented to nursing and social work researchers and representatives from the organisation, Nordic Standards for Children and Adolescents Rights and Needs in Health Care (NOBAB). They will be asked to review the findings for their implications.

### Patient and public involvement

Representatives from NOBAB will be involved as stakeholders and consulted throughout the research process. Children of parents diagnosed with cancer may be considered a vulnerable group, and participation would require ethical approval. Consequently, children were not involved in this protocol nor in the forthcoming scoping review, although their perspective would have been valuable.

## supplementary material

10.1136/bmjopen-2024-084240 online supplemental file 1
