# Supplementary material for: Children’s participation in the development, use and evaluation of support interventions for children of a parent diagnosed with cancer: a scoping review protocol
Source: BMJ Open. 2024 Aug 28;14(8):e084240. doi: 10.1136/bmjopen-2024-084240 (PMC11367290; doi:10.1136/bmjopen-2024-084240)
Supplement: online supplemental file 1 [file bmjopen-14-8-s001.pdf]

Supplemental file 1. Preliminary search in PubMed via NLM, November 2020

Population		Result
1	**"Child of Impaired Parents"[Mesh] OR "Adolescent"[Mesh] OR "Child"[Mesh]**	3,048,505
2	**child[tiab] OR children[tiab] OR youth*[tiab] OR offspring[tiab] OR adolescen*[tiab]**	1,576,343
3	1 OR 2	3,533,652
Concept		
4	**"Social Support"[Mesh] OR "Counseling"[Mesh]**	114,450
5	**Counsel*[tiab] OR support*[tiab]**	1,678,557
6	4 OR 5	1,722,512
7	**"Perception" osäker på denna term i kontrollerad form**	434,319
8	**help*[tiab] OR need*[tiab] OR experienc*[tiab] OR perception*[tiab]**	3,629,849
9	7 OR 8	3,937,891
10	6 AND 9	494,674
Context		
11	**"Neoplasms"[Mesh]**	3,382,016
12	**neoplasm*[tiab] OR cancer*[tiab] OR tumour*[tiab] OR tumor*[tiab] OR oncologic*[tiab] OR malign*[tiab] OR leukem*[tiab] OR cyst*[tiab]**	3,691,005
13	**11 OR 12**	4,650,186
14	**"Parents"[Mesh] OR "Parenting"[Mesh]**	124,665
15	**parent*[tiab] OR parental*[tiab] OR mother*[tiab] OR maternal*[tiab] OR father*[tiab] OR paternal*[tiab]**	813,981
16	13 OR 15	835,802
17	13 AND 16	70,608
Combined	3 AND 10 AND	2,300
