# Supplementary material for: Children’s participation in the development, use and evaluation of support interventions for children of a parent diagnosed with cancer: a scoping review protocol
Source: BMJ Open. 2024 Aug 28;14(8):e084240. doi: 10.1136/bmjopen-2024-084240 (PMC11367290; doi:10.1136/bmjopen-2024-084240)
Supplement: Supplementary file 2 [file bmjopen-2024-084240.reviewer_comments.pdf]

PEER REVIEW HISTORY

BMJ Open publishes all reviews undertaken for accepted manuscripts. Reviewers are asked to complete a checklist review form and are provided with free text boxes to elaborate on their assessment. These free text comments are reproduced below.

ARTICLE DETAILS

**TITLE (PROVISIONAL)**	CHILDREN´S PARTICIPATION IN THE DEVELOPMENT, USE AND EVALUATION OF SUPPORT INTERVENTIONS FOR CHILDREN OF A PARENT DIAGNOSED WITH CANCER: a scoping review protocol
**AUTHORS**	Samuelsson, Maria; Möllerberg, Marie-Louise; Edman, Kristina; Hansson, Kristofer; Enskär, Karin; Wennick, Anne

VERSION 1 - REVIEW

**REVIEWER NAME**	Høeg, Beverley Lim
**REVIEWER AFFILIATION**	Danish Cancer Society Research Center, Psychological Aspects of Cancer
**REVIEWER CONFLICT OF INTEREST**	None.
**DATE REVIEW RETURNED**	19-Feb-2024

**GENERAL COMMENTS**	Thank you for the opportunity to review this scoping review protocol. Patient and public involvement in research (PPI-R) is becoming increasingly important and I commend the authors for focusing on PPI-R among children. However, I have some comments below, which I hope can strengthen the reporting of this protocol and clarify the focus of study.<br><br>1. Abstract:<br><br>It would be helpful to define “patient participation in research,” as different terms are currently in use (I, myself, use PPI-R or “involvement” because the term “participant” often refers to the person who takes part in a study as a subject). It would also be helpful to state exactly what “gaps have been found” and explain why “children’s participation in research is central.” Currently, the authors state that this is because “in all situations that concern children, they have a right to have their voices heard,” which is a broad statement.<br><br>The authors also state that the aim of the review is to “map the existing literature on children’s participation in research,” but what exactly do they aim to map? Clarifying the knowledge gaps that this review aims to fill and what the expected benefits are of involving children in research will strengthen the rationale for this important study.<br><br>2. Introduction:<br><br>Similar comments as for the abstract.<br><br>The definition of “participation” currently given as a “feeling of belonging and engagement by the individual in relation to being

	active in a certain context" (line 101-103) is very broad and does not distinguish between the children who are participants in a research study as subjects (actually receiving the supportive interventions) and the children who are involved in the development of the supportive intervention as advisors or collaborators with the researchers (which I believe is what this scoping review is about, unless I am mistaken). Thus, the term "participation" and how it is being used in this review needs to be clarified.<br><br>Also, the authors mention "lack of participation in the research process" (line 106), but research consists of many parts (e.g. defining the research question, developing the intervention, advising on methods and data collection, analyzing data, etc.). It would be helpful for the authors to specify which part of the research process they believe that children should participate in and when/how children may safely and ethically be involved. For example, do the authors believe that children should be involved at any age? And why should researchers involve children in the first place – what are the benefits?<br><br>Note: The second sentence in the first paragraph (line 81) "For this study, parents are defined as biological parents or legal guardians" belongs in the method section.<br><br>3. Aim:<br><br>As mentioned for the abstract, the aim of this review needs to be clearly defined. What do the authors aim to map out? Is it just whether researchers involved children in developing the supportive intervention? Or it is also how children were involved, what age groups, the methods used and to what extent were their perspectives incorporated? A clear aim is important as as it will guide the screening and data extraction/charting steps later.<br><br><br>4. Methods:<br><br>The current research question and Participants, Concept, Context formulation (P=children, C=support, C= parental cancer) appears to be very broad and may make screening and data charting difficult. If the aim is to map PPI-R, then I would suggest narrowing the determinants, for example: P=children aged X to X years; C=participation in the research process in roles other than as a subject; C=development of support for children facing parental cancer.<br><br>The authors mention a conceptual framework that they have used to derive their research questions from (line 155). I believe that it would benefit the manuscript if this framework is already introduced in the Introduction section, and in more detail, to guide the reader as to what the focus of this review will be.<br><br>I also suggest using the framework to refine the data extraction form that the authors plan to use. Currently, the authors state that they plan to extract information on Context, Support characteristics, Support development, Support evaluation, Intended outcomes and Persons involved in the research process. This information does not appear sufficient to map the important aspects of PPI-R mentioned in the framework, e.g. degree of involvement, the forum of

	involvement, whether involvement made a difference in the decisions made, etc.<br><br>Finally, could the authors clarify how they plan to analyze the data? Currently, it appears that they plan to analyze the data three times in different ways, which seems a-bit confusing.<br><br><br>5. Patient and public involvement:<br><br>It is noteworthy that the authors state that children of parents diagnosed with cancer are considered a vulnerable group and were therefore not involved in this study due to the need for ethical approval. Would children then also be considered too vulnerable to be involved in the development of support initiatives? I do believe that older children or adolescents could contribute valuable perspectives to the topic of this review and if possible, I urge the authors to consider involving at least one relevant user to give feedback on either the topic in general or the results of this review. User feedback often surprises and provides perspectives that we researchers have not thought about!<br><br>Finally, this protocol would benefit from a Discussion/Conclusion section where the authors state the potential impact of their review, discuss the strengths/limitations of their methods and how their findings may be expected to move the field of children's participation in research forward.

**REVIEWER NAME**	Valizadeh, Fatemeh
**REVIEWER AFFILIATION**	Lorestan University of Medical Sciences Social Determinants of Health Research Center, Pediatrics Nursing
**REVIEWER CONFLICT OF INTEREST**	None.
**DATE REVIEW RETURNED**	25-May-2024

**GENERAL COMMENTS**	I am glad that I had the opportunity to read your valuable article. According to the content of the article, this scoping review protocol has appropriate approaches and the correct methodology for conducting scoping review research. The information about the research team, the steps of conducting the research, the selection criteria of the studies, the process of extracting information, analyzing the data, and reporting the results are fully and accurately given in the article.<br>In general, the article is written well and appropriate specialized terms and vocabulary are used. Among the strengths of the article, it can be mentioned that the logical and fluent structure in the presentation of information, the use of appropriate specialized terms, and the observance of grammar and spelling rules are observed.<br>The following suggestions may help in strengthening this research:<br>- The words: family, young adults, and sarcoma may also be used in the search strategy<br>- What is the reason for limiting search resources to the listed items? It is better to use other sources of information such as Embase, Web of Science, Cochrane CENTRAL,...

VERSION 1 – AUTHOR RESPONSE

Reviewer 1

Thank you for the comprehensive and careful review of our manuscript. Your criticism truly enhanced the manuscript.

#1<br><br>Abstract	It would be helpful to define “patient participation in research,” as different terms are currently in use (I, myself, use PPI-R or “involvement” because the term “participant” often refers to the person who takes part in a study as a subject).
#1<br>Response	Thank you for highlighting that this needs clarification. Please see change in red font line 111-113.<br><br>The rational for this study is the growing awareness of the importance of patient and public involvement in research (PPI-R) (1-4). Defined as research to being carried out “with” or “by” members of the public, rather than “to”, “about” or “for” them.
#2	It would also be helpful to state exactly what “gaps have been found” and explain why “children’s participation in research is central.” Currently, the authors state that this is because “in all situations that concern children, they have a right to have their voices heard,” which is a broad statement.
#2<br>Response	Of course. This has been addressed. Please see change in red font line 38-44.<br><br>At times of parental cancer, children´s health and wellbeing are at risk, which is why interventions to support these children have been developed. When developing such intervention engagement of the population under study in research is endorsed to enhance relevance of research questions and to enhance uptake and dissemination of the findings. Since no previous review has mapped the ways children participate in the development, use and evaluation of these support interventions, the focus of the upcoming scoping review is to identify gaps in the literature for guidance of future research.
#3	The authors also state that the aim of the review is to “map the existing literature on children’s participation in research,” but what exactly do they aim to map? Clarifying the knowledge gaps that this review aims to fill and what the expected benefits are of involving children in research will strengthen the rationale for this important study.
#3<br>Response	This has been addressed. Please see change in red font line 38-44 (response #2).
#4<br><br>Introduction	Similar comments as for the abstract.<br><br>The definition of “participation” currently given as a “feeling of belonging and engagement by the individual in relation to being active in a certain context” (line 101-103) is very broad and does not distinguish between the children who are participants in a research study as subjects (actually receiving the supportive interventions) and the children who are involved in the development of the

<table>
<tr><td></td><td>supportive intervention as advisors or collaborators with the researchers (which I believe is what this scoping review is about, unless I am mistaken). Thus, the term “participation” and how it is being used in this review needs to be clarified.</td></tr>
<tr><td>#4

Response</td><td>Thank you for highlighting that this needs clarification. This has been addressed. Please see changes in red font line 95-109.

Despite these comprehensive reviews, there are still unanswered questions regarding children’s participation in these interventions. Due to a paradigm shift in child health research, from a family-centered viewpoint to a child-centered viewpoint (8), this study focuses on the existing literature from a child-centered viewpoint. Namely, focuses children´s participation in the development, use and evaluations of the interventions i.e both in research processes and as study participants. In child health research children can participate indirect or direct, e.g. through being present or being engaged. In accordance, for this study, participation is defined in accordance with Eriksson and Granlund (9) as “a feeling of belonging and engagement experienced by the individual in relation to being active in a certain context” and divided into two dimensions: presence (i.e., physically being there) and engagement (i.e., expressions of involvement). Thus, the approaching scoping review will search to provide an overview of the ways children participates (direct or indirect) in the development, use and evaluation of the support interventions by mapping literature on support interventions for children of parental cancer including children as study participants.

Further, the research questions have been revised accordingly. Please see changes in red font line 164-169.

i. If, how and when do the children participate in the intervention development (direct or indirect e.g. by proxy)?
ii. How does the children use the intervention (direct or indirect e.g. by proxy)?
iii. If, how and when does children´s participate in evaluations of the intervention (direct or indirect e.g. by proxy)?</td></tr>
<tr><td>#5</td><td>Also, the authors mention “lack of participation in the research process” (line 106), but research consists of many parts (e.g. defining the research question, developing the intervention, advising on methods and data collection, analyzing data, etc.). It would be helpful for the authors to specify which part of the research process they believe that children should participate in and when/how children may safely and ethically be involved. For example, do the authors believe that children should be involved at any age?</td></tr>
<tr><td>#5

Response</td><td>Thank you for raising these important questions. We have clarified the focus of this study throughout the manuscript. Please see change in red font line 105-109.</td></tr>
</table>

	Thus, the approaching scoping review will search to provide an overview of the ways children participates (direct or indirect) in the development, use and evaluation of the support interventions by mapping literature on support interventions for children of parental cancer including children as study participants.<br><br>Regarding the questions of when/how and ethics, they are of course of relevance for any empirical child health research study. However, we do not find them in under the scope of this protocol, since it refers to the mapping of the children's participation as reported in the existing literature regardless of the above.
#6	And why should researchers involve children in the first place – what are the benefits?
#6 Response	Thank you for highlighting that this needs clarification, please see change in red font line 116-122.<br><br>Involving the population under study is endorsed to ensure that those with a lived experience of the phenomenon being researched – those who are the most affected by the research findings – are listened to and given the opportunity to participate in decision making in the research processes, for example, through the formulating and prioritizing of research questions and the strategies and timings for data collection (6). Reported benefits are improved recruitment and response and retention rates (7), more relevant research, and the enhanced uptake and dissemination of the findings (8).
#7	Note: The second sentence in the first paragraph (line 81) "For this study, parents are defined as biological parents or legal guardians" belongs in the method section.
#7 Response	This has been addressed. Please see change in red font, line 160-161.
#8 Aim:	As mentioned for the abstract, the aim of this review needs to be clearly defined. What do the authors aim to map out? Is it just whether researchers involved children in developing the supportive intervention? Or it is also how children were involved, what age groups, the methods used and to what extent were their perspectives incorporated? A clear aim is important as as it will guide the screening and data extraction/charting steps later.
#8 Response	We agree that the aim is critical which is why we have carefully considered our main review questions with regard to the scope of the study, the research field and the methodology. However, we have revised it for precision, in accordance with your recommendation. We hope that this together with the revised introduction will enhance the clarity of the study´s intention. Please see change in red font line 132-134.

	The purpose of the scoping review is to map the existing literature on children's participation in the development, use and evaluation of support interventions for children of a parent diagnosed with cancer.
#9<br><br>Methods:	The current research question and Participants, Concept, Context formulation (P=children, C=support, C= parental cancer) appears to be very broad and may make screening and data charting difficult. If the aim is to map PPI-R, then I would suggest narrowing the determinants, for example: P=children aged X to X years; C=participation in the research process in roles other than as a subject; C=development of support for children facing parental cancer.
#9<br><br>Response	Thank you for these suggestions which would be highly appropriate if we had only been interested in PPI-R as you say. Our PCC was determined with regard to our research question and after careful consideration based on preliminary searches of the research field, methodological recommendations (9) and decided in collaboration with a research librarian well versed in undertaking scoping reviews. The continued searches showed promising results using this mnemonic.
#10	The authors mention a conceptual framework that they have used to derive their research questions from (line 155). I believe that it would benefit the manuscript if this framework is already introduced in the Introduction section, and in more detail, to guide the reader as to what the focus of this review will be.
#10<br><br>Response	Thank you for bringing to our attention that this framework needs to be further explained. In fact, after getting more familiar with the research field, e.g sparse reports on PPI-R, we found that this framework would not work as intended. Thus, this has been removed.
#11	I also suggest using the framework to refine the data extraction form that the authors plan to use. Currently, the authors state that they plan to extract information on Context, Support characteristics, Support development, Support evaluation, Intended outcomes and Persons involved in the research process. This information does not appear sufficient to map the important aspects of PPI-R mentioned in the framework, e.g. degree of involvement, the forum of involvement, whether involvement made a difference in the decisions made, etc.
#11<br><br>Response	Again, we agree with the points you are making. We have added to the extraction form based on your recommendations and the revised aim and research questions of the current study. Please see changes in red font line 227-234.<br><br>i) Context<br>ii) Study characteristics<br>iii) Participant characteristics<br>iv) Intervention characteristics<br>v) Intervention development<br>vi) Use of the intervention<br>vii) Intervention evaluation<br>viii) Intended outcomes

#12	Finally, could the authors clarify how they plan to analyze the data? Currently, it appears that they plan to analyze the data three times in different ways, which seems a-bit confusing.
#12<br><br>Response	Thank you for highlighting that this appears confusing. This has been addressed. Please see change in red font line 244-248.<br><br>Extracted data will be analyzed using descriptive content analysis as outlined by Elo and Kyngäs (10), guided by specific research questions divided under the headings “Development”, “Use” and “Evaluation”. The result will be presented through a descriptive summary of the included articles and findings complemented with tables and figures when appropriate.
#13	5. Patient and public involvement:<br><br>It is noteworthy that the authors state that children of parents diagnosed with cancer are considered a vulnerable group and were therefore not involved in this study due to the need for ethical approval. Would children then also be considered too vulnerable to be involved in the development of support initiatives? I do believe that older children or adolescents could contribute valuable perspectives to the topic of this review and if possible, I urge the authors to consider involving at least one relevant user to give feedback on either the topic in general or the results of this review. User feedback often surprises and provides perspectives that we researchers have not thought about!
#13<br><br>Response	We completely agree on the significance of user feedback! As child health researchers we believe that most children can have a say. However, this review was not designed to include feedback from these children.<br><br>With regard to the question of children being too vulnerable to be involved in research: We find that highly relevant for any empirical study within this field (similar to #5), but we do not find it under the scope of this study since this aims to map the children’s, participation as reported in the existing literature. However, it could be of relevance for the discussion section of the scoping review, so thank you for making this point.
#14	Finally, this protocol would benefit from a Discussion/Conclusion section where the authors state the potential impact of their review, discuss the strengths/limitations of their methods and how their findings may be expected to move the field of children’s participation in research forward.
#14<br><br>Response	Thank you for this suggestion, this has been addressed. Please see change in red font line 257-264.<br><br>To prevent the well-known negative consequences of parental cancer in a child´s life, support needs to be relevant for the children´s needs and support preferences (11). For that purpose, child involvement and engagement in the development, use and evaluation of interventions are stressed (12). Mapping of the children´s participation in the existing literature in a research field does not provide evidence for which intervention to use, as a systematic review would. However, the upcoming

	scoping review allows for the mapping of a phenomenon in a research field which enables for identification of gaps in the literature which may guide the direction for future research.

Reviewer 2 Thank you for your careful review and encouraging words.

1	The words: family, young adults, and sarcoma may also be used in the search strategy
#1 Response	Thank you for these relevant suggestions. We will carefully consider applying these in the final search strategy. However, given the age span of our study being 0-18 years we have chosen not to include the concept “young adults”.
2	What is the reason for limiting search resources to the listed items? It is better to use other sources of information such as Embase, Web of Science, Cochrane.
#2 Response	Thank you for this relevant question and suggestions. Cochrane is used which we have added the manuscript. Web of Science will, in accordance with your suggestion, be searched to ensure no reference is missed. Please see changes in red font line 186, 189 and Figure 1. Given the research question, the search strategy, and databases, the research team and the research librarian decided not to include Embase.
